# Adiponectin and the risk of gastrointestinal cancers in East Asians: Mendelian randomization analysis

**DOI:** 10.1002/cam4.4735

**Published:** 2022-04-05

**Authors:** Hua Jiang, Daojun Hu, Jun Wang, Bo Zhang, Chiyi He, Jiyu Ning

**Affiliations:** ^1^ Department of Gastroenterology The First Affiliated Hospital of Wannan Medical College Wuhu City Anhui Province China; ^2^ Department of Clinical Laboratory Xinhua Hospital Affiliated to Shanghai Jiao Tong University School of Medicine Shanghai China; ^3^ The First Affiliated Hospital Hengyang Medical School, University of South China Hengyang City Hunan Province China

**Keywords:** adiponectin, East Asian, gastrointestinal cancer, Mendelian randomization

## Abstract

**Background:**

Adiponectin is an important adipocytokine and has been associated with the risks of gastrointestinal cancers (GICs). Mendelian randomization (MR) analysis is needed to assess the causal relationships between adiponectin and GICs.

**Methods:**

We retrieved the summary data of genome‐wide association studies for adiponectin and six types of GICs in East Asians. A series of quality control steps were performed to select the eligible genetic instrumental tools. Horizontal pleiotropy and between‐SNP heterogeneity were tested to choose the primary MR method. We also conducted sensitivity analyses to test the robustness of the main findings.

**Results:**

We detected neither heterogeneity nor horizontal pleiotropy for the eligible SNPs in all of the MR analyses. Inverse variance weighted (IVW) was therefore used as the primary method, and suggested that per 10% increase in log‐transformed adiponectin level was significantly associated with a decreased risk of gastric cancer (odds ratio [OR] = 0.88, 95% CI 0.81, 0.96), whereas with an increased risk of hepatocellular carcinoma (OR = 1.26, 95% CI 1.09, 1.44) and of biliary tract cancer (OR = 1.54, 95% CI 1.12, 2.12). However, only the association between adiponectin and HCC risk was statistically significant after correction for multiple testing. No statistically significant association was detected between adiponectin and esophageal (OR = 1.05, 95% CI 0.89, 1.23), pancreatic (OR = 1.04, 95% CI 0.78, 1.37), and colorectal cancers (OR = 1.00, 95% CI 0.93, 1.07). Sensitivity analyses did not find contradictory results.

**Conclusion:**

High level of adiponectin may have a causal effect on and can serve as a biomarker for the carcinogenesis of gastric cancer, hepatocellular carcinoma, and biliary tract cancer.

## Graphical abstract

Genetically determined level of adiponectin was associated with an increased risk of hepatocellular carcinoma and biliary tract cancer, whereas with a decreased risk of gastric cancer. No significant association was detected between adiponectin and risks of esophageal, pancreatic, colorectal cancers.

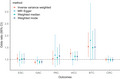

## 1 INTRODUCTION

Gastrointestinal cancers (GICs), mainly including esophageal, gastric, pancreatic, liver, gallbladder, and colorectal cancers, are commonly diagnosed in East Asians and impose an increasing disease burden.^1^, ^2^ The risk factors for GICs have been widely investigated and are shared to a great degree.^3^ Although enormous efforts have been made to combat GICs, there was still a long way to reduce the disease burden of GICs. One of the key points is early screening (and/or diagnosis) based on circulating biomarkers.

Adiponectin is one of the most important adipocytokines secreted by adipocytes and is deemed to play an important role in the carcinogenesis of GICs.^4^ Previous epidemiological studies reported that the level of plasma adiponectin was associated with the risk of several types of GICs. For example, population‐based studies reported that a low plasma adiponectin level was associated with an increased risk for gastric cancer (GAC), pancreatic cancer (PAC), and esophageal cancer (ESC).^5^, ^6^, ^7^ On the contrary, in a nested case–control study, Aleksandrova et al. found that elevated level of adiponectin was associated with an increased risk of hepatocellular carcinoma (HCC).^8^ The correlation between blood adiponectin and colorectal cancer (CRC) was inconsistent between studies.^9^

The findings from observational studies might be biased by several factors, such as incomplete adjustment for confounders, transient fluctuation of blood biomarker, and small sample size, and might subject to reverse causality. Mendelian randomization (MR) analysis that implemented genetic information is less susceptible to the aforementioned shortcomings because alleles are randomly assigned during meiosis and germline genetic variants are unaffected by environmental confounders.^10^, ^11^ Along with the accumulation of genetic data from genome‐wide association study (GWAS), MR analysis has been widely used to detect causal relationships between exposures and outcomes.^12^, ^13^, ^14^, ^15^ For instance, Cornish et al. reported a nonsignificant correlation between circulating adiponectin and CRC risk.^16^ The similar results were also reported by Nimptsch et al.^17^ Dimou et al. found an inverse association between adiponectin and risk of CRC, while no association was found for adiponectin and risk of PAC.^18^ The aforementioned studies were conducted among Europeans. However, the relationships between circulating adiponectin and GICs in East Asians have not been evaluated by MR. In the present study, based on genetic information from East Asians and leveraging MR methods, we aimed to assess the causal relationships between adiponectin and six types of GICs, that are ESC, GAC, PAC, HCC, biliary tract cancer (BTC), and CRC.

## 2 METHODS

### 2.1 GWAS of adiponectin

We retrieved the GWAS summary statistics of adiponectin from Asian Genetic Epidemiology Network (AGEN), which conducted a meta‐analysis of GWAS for adiponectin in 7827 individuals of East Asian ancestry.^19^ The details of the GWAS for adiponectin were shown in Wu et al.^19^ Briefly, the GWAS consisted of 7827 Chinese, Korean, and Filipino individuals from SP2, the Korean Cancer Prevention Study II (KCPS‐II), CLHNS and the Nutrition and Health of Aging Population in China (NHAPC). Within each study, adiponectin was natural logarithm transformed to approximate normal distribution. Multivariable linear regression models assuming an additive mode of inheritance were applied to test for association with genotyped or imputed SNPs by accounting for age, sex, and body mass index (BMI). In the meta‐analysis of GWAS, approximately 2.5 million SNPs were used by an inverse variance weighted method implemented in METAL.^19^ The proportion of variation in adiponectin that explained by SNPs was 2.65%.

### 2.2 GWAS of gastrointestinal cancers

To ensure the comparability in participants' ancestry, we retrieved the GWAS summary data of the six types GICs from BioBank Japan (BBJ)^20^ via IEU open GWAS project (https://gwas.mrcieu.ac.uk/). BBJ is a prospective biobank that collaboratively collected DNA and serum samples from 12 medical institutions in Japan and recruited ~200,000 participants, mainly of Japanese ancestry.^20^ The BBJ participants were genotyped with the Illumina HumanOmniExpressExome BeadChip or a combination of the Illumina HumanOmniExpress and HumanExome BeadChip. Quality control of participants and genotypes was performed as described elsewhere.^21^ The numbers of case and controls and SNPs used in the GWAS for GICs are shown in Table S1. A generalized linear mixed model implemented in SAIGE (v.0.37) was applied to perform the GWAS, in which age, age^2^, sex, age × sex, age^2^ × sex, and the top 20 principal components were adjusted. In IEU open GWAS platform, the GWAS id corresponding to ESC, GAC, PAC, HCC, BTC, and CRC was “bbj‐a‐117”, “bbj‐a‐119”, “bbj‐a‐140”, “bbj‐a‐158”, “bbj‐a‐92”, and “bbj‐a‐107”, respectively.

### 2.3 Genetic instrumental variables and GWAS summary statistics

We conducted a series of quality control steps to select eligible instrumental SNPs as performed elsewhere.^13^ First, we extracted the SNPs that reached the *p*‐value threshold (*p* < 5 × 10^−8^) in the GWAS of adiponectin. Second, we assessed the linkage disequilibrium (LD) between SNPs based on the East Asians from the 1000 genomes project and then performed a clumping process (*R* ^2^ < 0.1, window size = 10,000 kb). Among those pairs of SNPs that had a LD estimate above the specified threshold (0.1), only the SNP with the lower *p*‐value would be retained. Third, we removed SNPs with a minor allele frequency <5%.

We then extracted the GWAS summary statistics including beta coefficient and standard error of the eligible SNPs from GWAS of adiponectin and of the GICs. For instrumental SNPs that absent in the GWAS of GICs, we retrieved the data of a SNP proxy that had LD estimate *R* ^2^ > 0.9 with the requested SNP. The effects of ambiguous SNPs with inconsistent alleles and palindromic SNPs with ambiguous strand were either corrected or directly excluded in the subsequent two‐sample MR analysis.

### 2.4 Mendelian randomization estimates

**FIGURE 1:**
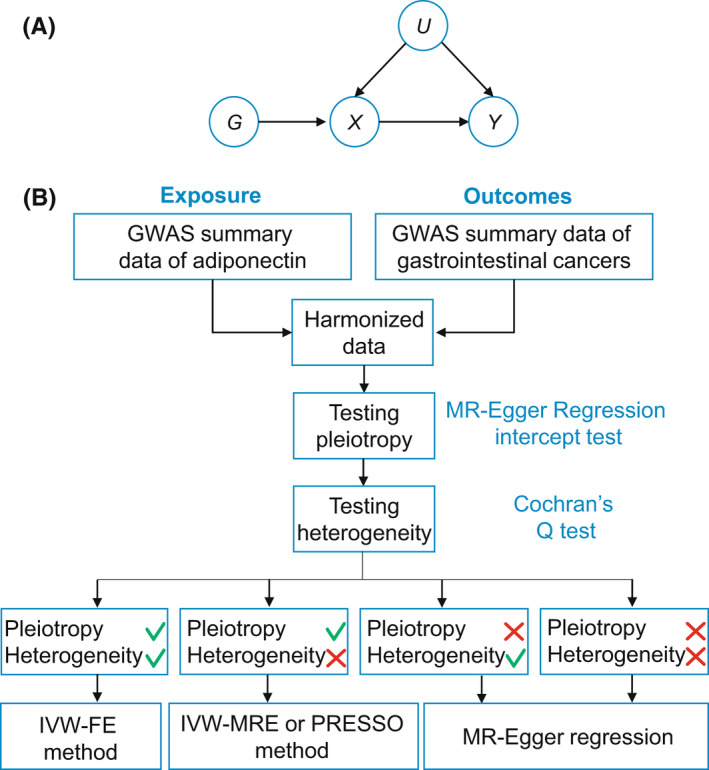
Mendelian randomization (MR) analysis used in this study. (A) The schematic representation of MR analysis. G, X, Y, and U Represents genetic instrumental tools, exposure of interest, outcome(s) of interest, and unobserved confounders, respectively. (B) The flowchart of MR analysis. The green √ means passing the test and the red × means not passing the test. FE, fixed effect; MRE, multiplicative random effects

The schematic representation of MR analysis is shown in Figure 1A and the methodological details have been shown in previous studies.^11^, ^22^ As displayed in Figure 1B, we constructed a flowchart to conduct MR step by step. First, we harmonized the GWAS summary data of adiponectin and of GICs using the selected SNP as matching index. Second, we used MR‐Egger regression to test the horizontal pleiotropy. Third, we used Cochran's *Q* test in inverse variance weighted (IVW) method and MR‐Egger regression to detect the between‐SNP heterogeneity. We selected the primary MR method as follows:

1. if neither horizontal pleiotropy nor heterogeneity was detected, use fixed‐effect IVW.
2. if no horizontal pleiotropy but heterogeneity, use IVW with multiplicative random effect or MR‐PRESSO method.^23^
3. if horizontal pleiotropy was detected, use MR‐Egger regression.^24^

We also checked the consistency of the directions in all four MR methods (i.e., IVW, MR‐Egger regression, weighted median, and weighted mode methods). Finally, we conducted a leave‐one‐out analysis to detect the influential SNP. All estimates of MR analyses were multiplied by 1.1 and then converted to odds ratio (OR) to quantify the risk of GICs per 10% increase in log‐transformed level of adiponectin. *F*‐statistics was used to assess the strength of relationship between instrumental variables and phenotype, and calculated using the following Equation^25^:

(1) $$F=\frac{R^{2}/k}{\left(1-R^{2}\right)/\left(n-k-1\right)},$$

where *R* ^2^ is the proportion of adiponectin variance, *k* is the number of instruments used in the model, and *n* is the sample size. We used the *I* ^2^‐GX statistic that calculated from MR‐Egger regression model to estimate the potential relative (dilution) bias due to measurement error.^26^ All statistical analyses were implemented using TwoSampleMR and MRPRESSO packages in R program (v 4.1.1). We calculated the statistical power for MR analyses using *mRnd* website (https://shiny.cnsgenomics.com/mRnd/).^27^ *p* < 0.05 was considered statistically significant.

## 3 RESULTS

### 3.1 Validity of the instrumental variables

After quality control process, we included eight eligible SNPs in this analysis (Table S2). The mean F‐statistics for the eight SNPs was 92.9, which satisfied the threshold of *F* > 10, typically recommended for MR analyses. The *I* ^2^‐GX value was 96.9%, which was >95%, indicating a low probability of weak instrument bias and an acceptable collective suitability of instrumental variables for MR analyses. As shown in Table 1, we detected neither between‐SNP heterogeneity nor horizontal pleiotropy for the eligible SNPs in all of the MR analyses. These findings suggest the validity of the selected instrumental variables and fixed‐effect IVW method was therefore used as the primary method.

**TABLE 1:** Statistics of testing for heterogeneity and pleiotropy in Mendelian randomization analysis

Outcomes	Between‐SNP heterogeneity^a^		Horizontal pleiotropy		Odds ratio (95% CI)^b^	*p*‐value	Statistical power to identify OR >1.1 or <0.9^c^	Statistical power to identify OR >1.3 or <0.7^c^
	*Q* statistics	*p*‐value	Egger intercept	*p*‐value				
Esophageal cancer	4.11	0.662	0.015	0.658	1.05 (0.89, 1.23)	0.631	0.10	0.42
Gastric cancer	8.02	0.237	−0.014	0.419	0.88 (0.81, 0.96)	0.009	0.25	0.97
Pancreatic cancer	2.86	0.826	−0.027	0.629	1.04 (0.78, 1.37)	0.823	0.06	0.18
Hepatocellular carcinoma	3.70	0.718	0.047	0.125	1.26 (1.09, 1.44)	0.003	0.11	0.56
Biliary tract cancer	5.22	0.516	−0.032	0.615	1.54 (1.12, 2.12)	0.016	0.06	0.15
Colorectal cancer	6.91	0.329	0.0003	0.983	1.00 (0.93, 1.07)	0.956	0.26	0.98

a Heterogeneity was tested by MR‐Egger regression. The results from inverse variance weighted method were similar to that of the MR‐Egger method, and therefore were not shown here.

^b^ Odds ratios were derived from fixed‐effect inverse variance weighted method.

^c^ OR = odds ratio per genetically predicted SD unit increase in risk factor.

### 3.2 Causal relationships between adiponectin and gastrointestinal cancers

According to the IVW method, we found that per 10% increase in log‐transformed adiponectin level was significantly associated with a decreased risk of GAC (OR = 0.88, 95% CI 0.81, 0.96), whereas with an increased risk of HCC (OR = 1.26, 95% CI 1.09, 1.44) and of BTC (OR = 1.54, 95% CI 1.12, 2.12) (Table 1). For the other three types of GICs, no statistically significant association was detected (Table 1). Of note only the association between adiponectin and HCC risk was statistically significant at the threshold of 0.0083 (adjusted *p*‐value: 0.05/6). The MR analyses might be under‐powered (<80%) in the current scenario (Table 1).

**FIGURE 2:**
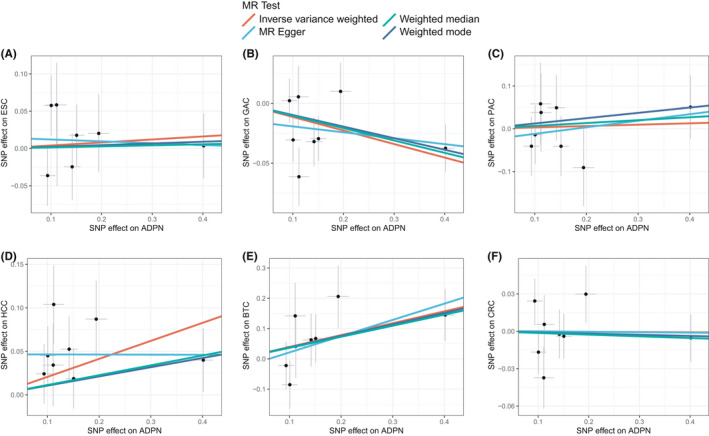
The scatter plots of associations between adiponectin (ADPN) and gastrointestinal cancers. The dots represent SNPs used in this analysis and the bars denote the 95% confidence intervals. (A) esophageal cancer (B) gastric cancer (C) pancreatic cancer (D) hepatocellular carcinoma (E) biliary tract cancer (F) colorectal cancer

**FIGURE 3:**
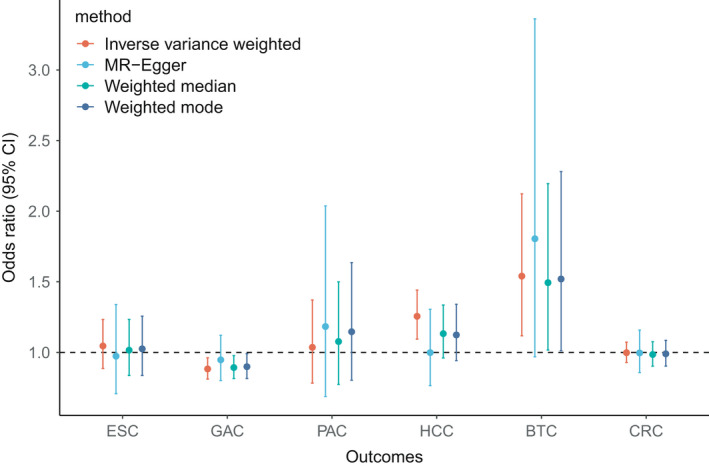
The associations between adiponectin and gastrointestinal cancers according to different Mendelian randomization methods. ESC, esophageal cancer; GAC, gastric cancer; PAC, pancreatic cancer; HCC, hepatocellular carcinoma; BTC, biliary tract cancer; CRC, colorectal cancer

We conducted sensitivity analyses to check the robustness of the IVW estimates. As shown in Figure 2 and Table S3, the MR estimates calculated from the four different methods were highly consistent in terms of the direction. For GAC and BTC, the significant association with adiponectin was also detected in weighted median and weighted mode methods (Figure 3; Table S3). The significant association between adiponectin and HCC was only found in IVW method whereas not in the other three methods. For the rest three GICs, that are ESC, PAC, and CRC, all of the methods did not detect a significant correlation with adiponectin (Figure 3; Table S3).

Figures S1–S6 display the results of leave‐one‐out analysis for each GICs. The associations between adiponectin and GAC and HCC remained significant when excluding any one of the SNPs (Figures S2 and S4). However, the association of adiponectin with BTC was not statistically significant when leaving out the rs12051272 or rs11646213 (Figure S5). There was still no significant association of adiponectin with ESC, PAC, and CRC to be detected in this analysis.

## 4 DISCUSSION

In the present study, we established a causal relationship of circulating adiponectin with the risk of GAC, HCC, and BTC leveraging MR analysis. Briefly, we reported that an elevated level of adiponectin was associated with a decreased risk of GAC, whereas with increased risks of HCC and BTC. We did not detect causal relationships between adiponectin and ESC, PAC, and CRC. These findings were to some extent robust due to the following reasons: (1) suitable genetic instrumental tools (F‐statistics >10 and *I* ^2^‐GX > 95%); (2) no horizontal pleiotropy and between‐SNP heterogeneity was detected; (3) results calculated from the four methods were highly consistent, albeit the nuances in OR estimates; and (4) influential SNP was not detected in leave‐one‐out analysis for GAC and HCC, but for BTC.

The six major types of GICs were demonstrated to closely link with adiposity, and the risk of GICs were increased with the increment of BMI to a different degree.^28^ However, debates still remained for this issue. Adiponectin inversely correlates with BMI and visceral adiposity.^4^, ^29^ The associations of adiponectin with GICs have been reported in previous studies, although the literature data were sometimes conflicting. Some studies reported that adiponectin might confer a protective effect on the development and progression of GICs due to its features of anti‐inflammation and anti‐proliferation.^5^, ^29^, ^30^, ^31^, ^32^ On the other hand, there were evidences from population‐based studies suggested no correlation between adiponectin and the risk of GICs^9^, ^33^, ^34^ and that adiponectin conferred an increased risk for liver cancer.^35^, ^36^ The inconsistent findings of the observational studies indicated a putative role of adiponectin in the carcinogenesis of GICs and greatly justified the necessity of our MR study. From the methodological perspective, MR analysis is less susceptible to confounders and inverse causality than observational study. We could draw a relatively robust conclusion if the MR assumptions are completely met.

In our study, we found that a low plasma adiponectin level might be causally associated with an increased risk for GAC. This finding was consistent with that of some epidemiological studies,^5^, ^37^ of which most were cross‐sectional designs. On the contrary, we found that a high level of adiponectin might be causally related with an increased risk of HCC, which shares many risk factors with GAC. This result was highly in line with that of previous epidemiological studies,^8^, ^36^, ^38^ indicating that adiponectin could serve as a risk factor for HCC, and more importantly, that GAC and HCC were distinct in physiopathologic process. Of note is that BMI was adjusted in the GWAS of adiponectin, suggesting the putative causal relationships between adiponectin and risks of GAC and HCC might be driven by mechanisms that were independent of obesity, and indicating multiple biological roles of adiponectin.

The protective effect of adiponectin was mainly related to its indirect antineoplastic actions, including insulin‐sensitizing, immune‐related, and angiogenesis‐related effects.^39^ For example, adiponectin has been demonstrated to inhibit B cells differentiation from bone marrow and elevate expression of a set of anti‐inflammatory cytokines including IL‐10, IL‐6, TNF‐α, and IFN‐γ.^40^ Moreover, adiponectin was deemed to be a strong inhibitor of the PI3K/Akt/mTOR pathway, thus limiting the tumor cell growth induced by insulin and by other growth factors.^41^ Other underlying mechanisms have also been proposed.^4^ On the other hand, the positive association between adiponectin and HCC risk is surprising and needs further investigations, given that adiponectin was reported to be protective against fatty liver disease and a low circulating adiponectin has been observed in patients with chronic hepatitis and hepatic steatosis.^42^, ^43^ However, Hui et al. reported that serum adiponectin levels were elevated in patients with advanced liver fibrosis.^44^ Since adiponectin is majorly metabolized in the liver, circulating adiponectin may represent a biomarker of liver fibrosis, as it has been reported to negatively associated with the platelet count.^38^ Moreover, elevated plasma adiponectin level has been associated with inflammatory diseases, such as arthritis, preeclampsia, and end‐stage renal disease,^45^, ^46^, ^47^ suggesting adiponectin may also have pro‐inflammatory roles.

We also detected a positive association between adiponectin and the risk of BTC, which shares multiple aspects with HCC in terms of histological features, activation of pathways linked to disease development, and prognosis. However, this association disappeared in the leave‐one‐out analysis when excluding rs12051272 or rs11646213. The two polymorphisms are located in *CDH13* gene, which encodes a member of the cadherin superfamily. Previous studies reported that the two variants were associated with a set of traits, such as serum lipid levels, risk of hypertension and non‐small cell lung cancer, and preeclampsia.^48^, ^49^, ^50^, ^51^ However, there was no evidence linked these variants to BTC. Future studies are warranted to further investigate the role of *CDH13* gene in the genesis of BTC.

The limitations of our study should be noted. First, we focused on study participants of East Asians ancestry, thus limiting the extrapolation of our findings to other populations. Second, the histological subtypes of GICs, such as esophageal squamous cell carcinoma and esophageal adenocarcinoma, were not considered in our study, and might bias the MR estimates because of the differences between cancer subtypes in many ways. Third, adiponectin exerts biological roles depending on its receptors, AdipoR1 and *R* ^2^, which might influence the association of adiponectin with diseases. Finally, due to the small proportions of the six types of GICs, our MR estimates might be under‐powered. Future studies with larger sample size for cancer cases were warranted.

In summary, in the current MR study, we found that high level of adiponectin was associated with a decreased risk of GAC, whereas with increased risks of HCC and BTC. Adiponectin could serve as a biomarker for GAC, HCC, and BTC in clinical practice. Moreover, the heterogeneous associations between adiponectin and GICs connote a complex role of this adipocytokine in the onset of GICs. More investigations that pinpoint the underlying mechanisms are warranted.

## CONFLICT OF INTEREST

The authors declare that they have no competing interest.

## AUTHOR CONTRIBUTIONS

JN, CH, and HJ conceived the idea for the study. HJ and DH obtained the genetic data. HJ, BZ, and DH performed the data analyses. JN, CH, WJ, and HJ interpreted the results of the data analyses. HJ wrote the manuscript. All authors read and approved the final manuscript.

## CONSENT FOR PUBLICATION

Not applicable.

## ETHICS STATEMENT

This study was based on publicly available data and the ethics approvals are available in the original studies. All methods were carried out in accordance with relevant guidelines and regulations.

## Supporting information


Appendix S1

## Data Availability

The datasets generated and/or analyzed during the current study are available in the following repository: GWAS summary data of adiponectin were available on the Asian Genetic Epidemiology Network website (AGEN; https://blog.nus.edu.sg/agen/). GWAS summary data of gastrointestinal cancer were available on IEU Open GWAS project website (https://gwas.mrcieu.ac.uk/).

## References

[1] Sung H , Ferlay J , Siegel RL , et al. Global cancer statistics 2020: GLOBOCAN estimates of incidence and mortality worldwide for 36 cancers in 185 countries. CA Cancer J Clin. 2021;71(3):209‐249. 33538338 10.3322/caac.21660

[2] Fitzmaurice C , Abate D , Abbasi N , et al. Global, regional, and National Cancer Incidence, mortality, years of life lost, years lived with disability, and disability‐adjusted life‐years for 29 cancer groups, 1990 to 2017: a systematic analysis for the global burden of disease study. JAMA Oncol. 2019;5(12):1749‐1768. 31560378 10.1001/jamaoncol.2019.2996 PMC6777271

[3] Ulrich CM , Himbert C , Holowatyj AN , Hursting SD . Energy balance and gastrointestinal cancer: risk, interventions, outcomes and mechanisms. Nat Rev Gastroenterol Hepatol. 2018;15(11):683‐698. 30158569 10.1038/s41575-018-0053-2 PMC6500387

[4] Parida S , Siddharth S , Sharma D . Adiponectin obesity, and cancer: clash of the bigwigs in health and disease. Int J Mol Sci. 2019;20(10):2519. 10.3390/ijms20102519 PMC6566909 31121868

[5] Ishikawa M , Kitayama J , Kazama S , Hiramatsu T , Hatano K , Nagawa H . Plasma adiponectin and gastric cancer. Clin Cancer Res. 2005;11(2 Pt 1):466‐472. 15701829

[6] Bao Y , Giovannucci EL , Kraft P , et al. A prospective study of plasma adiponectin and pancreatic cancer risk in five US cohorts. J Natl Cancer Inst. 2013;105(2):95‐103. 23243202 10.1093/jnci/djs474 PMC3545904

[7] Yildirim A , Bilici M , Cayir K , Yanmaz V , Yildirim S , Tekin SB . Serum adiponectin levels in patients with esophageal cancer. Jpn J Clin Oncol. 2009;39(2):92‐96. 19116211 10.1093/jjco/hyn143

[8] Aleksandrova K , Boeing H , Nöthlings U , et al. Inflammatory and metabolic biomarkers and risk of liver and biliary tract cancer. Hepatology. 2014;60(3):858‐871. 24443059 10.1002/hep.27016 PMC4231978

[9] Otani K , Ishihara S , Yamaguchi H , et al. Adiponectin and colorectal cancer. Surg Today. 2017;47(2):151‐158. 27061803 10.1007/s00595-016-1334-4

[10] Koellinger PD , de Vlaming R . Mendelian randomization: the challenge of unobserved environmental confounds. Int J Epidemiol. 2019;48(3):665‐671. 31263889 10.1093/ije/dyz138 PMC6659461

[11] Burgess S , Davey Smith G , Davies NM , et al. Guidelines for performing Mendelian randomization investigations. Wellcome Open Res. 2019;4:186. 32760811 10.12688/wellcomeopenres.15555.1 PMC7384151

[12] Ference BA , Ray KK , Catapano AL , et al. Mendelian randomization study of ACLY and cardiovascular disease. N Engl J Med. 2019;380(11):1033‐1042. 30865797 10.1056/NEJMoa1806747 PMC7612927

[13] Wu F , Huang Y , Hu J , Shao Z . Mendelian randomization study of inflammatory bowel disease and bone mineral density. BMC Med. 2020;18(1):312. 33167994 10.1186/s12916-020-01778-5 PMC7654011

[14] Mohammadi‐Shemirani P , Chong M , Pigeyre M , Morton RW , Gerstein HC , Paré G . Effects of lifelong testosterone exposure on health and disease using Mendelian randomization. Elife. 2020;9:e58914. 33063668 10.7554/eLife.58914 PMC7591257

[15] Ference BA , Yoo W , Alesh I , et al. Effect of long‐term exposure to lower low‐density lipoprotein cholesterol beginning early in life on the risk of coronary heart disease: a Mendelian randomization analysis. J Am Coll Cardiol. 2012;60(25):2631‐2639. 23083789 10.1016/j.jacc.2012.09.017

[16] Cornish AJ , Law PJ , Timofeeva M , et al. Modifiable pathways for colorectal cancer: a mendelian randomisation analysis. Lancet Gastroenterol Hepatol. 2020;5(1):55‐62. 31668584 10.1016/S2468-1253(19)30294-8 PMC7026696

[17] Nimptsch K , Song M , Aleksandrova K , et al. Genetic variation in the ADIPOQ gene, adiponectin concentrations and risk of colorectal cancer: a Mendelian randomization analysis using data from three large cohort studies. Eur J Epidemiol. 2017;32(5):419‐430. 28550647 10.1007/s10654-017-0262-y PMC5535815

[18] Dimou NL , Papadimitriou N , Mariosa D , et al. Circulating adipokine concentrations and risk of five obesity‐related cancers: a Mendelian randomization study. Int J Cancer. 2021;148(7):1625‐1636. 33038280 10.1002/ijc.33338 PMC7894468

[19] Wu Y , Gao H , Li H , et al. A meta‐analysis of genome‐wide association studies for adiponectin levels in east Asians identifies a novel locus near WDR11‐FGFR2. Hum Mol Genet. 2014;23(4):1108‐1119. 24105470 10.1093/hmg/ddt488 PMC3900106

[20] Sakaue S , Kanai M , Tanigawa Y , et al. A cross‐population atlas of genetic associations for 220 human phenotypes. Nat Genet. 2021;53(10):1415‐1424. 34594039 10.1038/s41588-021-00931-x PMC12208603

[21] Akiyama M , Ishigaki K , Sakaue S , et al. Characterizing rare and low‐frequency height‐associated variants in the Japanese population. Nat Commun. 2019;10(1):4393. 31562340 10.1038/s41467-019-12276-5 PMC6764965

[22] Pierce BL , Ahsan H , Vanderweele TJ . Power and instrument strength requirements for Mendelian randomization studies using multiple genetic variants. Int J Epidemiol. 2011;40(3):740‐752. 20813862 10.1093/ije/dyq151 PMC3147064

[23] Verbanck M , Chen CY , Neale B , Do R . Detection of widespread horizontal pleiotropy in causal relationships inferred from Mendelian randomization between complex traits and diseases. Nat Genet. 2018;50(5):693‐698. 29686387 10.1038/s41588-018-0099-7 PMC6083837

[24] Burgess S , Thompson SG . Interpreting findings from Mendelian randomization using the MR‐egger method. Eur J Epidemiol. 2017;32(5):377‐389. 28527048 10.1007/s10654-017-0255-x PMC5506233

[25] Zhu G , Zhou S , Xu Y , et al. Mendelian randomization study on the causal effects of omega‐3 fatty acids on rheumatoid arthritis. Clin Rheumatol. 2022. 10.1007/s10067-022-06052-y 35000008

[26] Bowden J , Del Greco MF , Minelli C , Davey Smith G , Sheehan NA , Thompson JR . Assessing the suitability of summary data for two‐sample Mendelian randomization analyses using MR‐egger regression: the role of the *I* ^2^ statistic. Int J Epidemiol. 2016;45(6):1961‐1974. 27616674 10.1093/ije/dyw220 PMC5446088

[27] Brion MJ , Shakhbazov K , Visscher PM . Calculating statistical power in Mendelian randomization studies. Int J Epidemiol. 2013;42(5):1497‐1501. 24159078 10.1093/ije/dyt179 PMC3807619

[28] Murphy N , Jenab M , Gunter MJ . Adiposity and gastrointestinal cancers: epidemiology, mechanisms and future directions. Nat Rev Gastroenterol Hepatol. 2018;15(11):659‐670. 29970888 10.1038/s41575-018-0038-1

[29] Barb D , Pazaitou‐Panayiotou K , Mantzoros CS . Adiponectin: a link between obesity and cancer. Expert Opin Investig Drugs. 2006;15(8):917‐931. 10.1517/13543784.15.8.917 16859394

[30] Fujisawa T , Endo H , Tomimoto A , et al. Adiponectin suppresses colorectal carcinogenesis under the high‐fat diet condition. Gut. 2008;57(11):1531‐1538. 18676419 10.1136/gut.2008.159293 PMC2582344

[31] Jiang J , Fan Y , Zhang W , et al. Adiponectin suppresses human pancreatic cancer growth through attenuating the β‐catenin signaling pathway. Int J Biol Sci. 2019;15(2):253‐264. 30745818 10.7150/ijbs.27420 PMC6367542

[32] Zhang R , Wu J , Liu D , Shan H , Zhang J . Anti‐inflammatory effect of full‐length adiponectin and proinflammatory effect of globular adiponectin in esophageal adenocarcinoma cells. Oncol Res. 2013;21(1):15‐21. 24330848 10.3727/096504013X13786659070235

[33] Kadri Colakoglu M , Bostanci EB , Ozdemir Y , et al. Roles of adiponectin and leptin as diagnostic markers in pancreatic cancer. Bratisl Lek Listy. 2017;118(7):394‐398. 28766348 10.4149/BLL_2017_077

[34] Joshi RK , Kim WJ , Lee SA . Association between obesity‐related adipokines and colorectal cancer: a case‐control study and meta‐analysis. World J Gastroenterol. 2014;20(24):7941‐7949. 24976730 10.3748/wjg.v20.i24.7941 PMC4069321

[35] Michikawa T , Inoue M , Sawada N , et al. Plasma levels of adiponectin and primary liver cancer risk in middle‐aged Japanese adults with hepatitis virus infection: a nested case‐control study. Cancer Epidemiol Biomarkers Prev. 2013;22(12):2250‐2257. 24045928 10.1158/1055-9965.EPI-13-0363

[36] Zhang L , Yuan Q , Li M , Chai D , Deng W , Wang W . The association of leptin and adiponectin with hepatocellular carcinoma risk and prognosis: a combination of traditional, survival, and dose‐response meta‐analysis. BMC Cancer. 2020;20(1):1167. 33256658 10.1186/s12885-020-07651-1 PMC7708253

[37] Nakajima TE , Yamada Y , Hamano T , et al. Adipocytokine levels in gastric cancer patients: resistin and visfatin as biomarkers of gastric cancer. J Gastroenterol. 2009;44(7):685‐690. 19430715 10.1007/s00535-009-0063-5

[38] Arano T , Nakagawa H , Tateishi R , et al. Serum level of adiponectin and the risk of liver cancer development in chronic hepatitis C patients. Int J Cancer. 2011;129(9):2226‐2235. 21170963 10.1002/ijc.25861

[39] Tumminia A , Vinciguerra F , Parisi M , et al. Adipose tissue, obesity and Adiponectin: role in endocrine cancer risk. Int J Mol Sci. 2019;20(12):2863. 10.3390/ijms20122863 PMC6628240 31212761

[40] Wolf AM , Wolf D , Rumpold H , Enrich B , Tilg H . Adiponectin induces the anti‐inflammatory cytokines IL‐10 and IL‐1RA in human leukocytes. Biochem Biophys Res Commun. 2004;323(2):630‐635. 15369797 10.1016/j.bbrc.2004.08.145

[41] Sengupta S , Peterson TR , Sabatini DM . Regulation of the mTOR complex 1 pathway by nutrients, growth factors, and stress. Mol Cell. 2010;40(2):310‐322. 20965424 10.1016/j.molcel.2010.09.026 PMC2993060

[42] Kim YS , Lee SH , Park SG , et al. Low levels of total and high‐molecular‐weight adiponectin may predict non‐alcoholic fatty liver in Korean adults. Metabolism: Clinical and Experimental. 2020;103(154026):154026. 31765666 10.1016/j.metabol.2019.154026

[43] Jonsson JR , Moschen AR , Hickman IJ , et al. Adiponectin and its receptors in patients with chronic hepatitis C. J Hepatol. 2005;43(6):929‐936. 16139921 10.1016/j.jhep.2005.05.030

[44] Hui CK , Zhang HY , Lee NP , et al. Serum adiponectin is increased in advancing liver fibrosis and declines with reduction in fibrosis in chronic hepatitis B. J Hepatol. 2007;47(2):191‐202. 17462782 10.1016/j.jhep.2007.02.023

[45] Choi HM , Doss HM , Kim KS . Multifaceted physiological roles of Adiponectin in inflammation and diseases. Int J Mol Sci. 2020;21(4):1219. 10.3390/ijms21041219 PMC7072842 32059381

[46] Eleuterio NM , Palei AC , Rangel Machado JS , Tanus‐Santos JE , Cavalli RC , Sandrim VC . Relationship between adiponectin and nitrite in healthy and preeclampsia pregnancies. Clin Chim Acta. 2013;423:112‐115. 23643962 10.1016/j.cca.2013.04.027

[47] Martinez Cantarin MP , Waldman SA , Doria C , et al. The adipose tissue production of adiponectin is increased in end‐stage renal disease. Kidney Int. 2013;83(3):487‐494. 23283133 10.1038/ki.2012.421 PMC3587362

[48] Li Y , Li C , Ma Q , et al. Genetic variation in CDH13 gene was associated with non‐small cell lung cancer (NSCLC): a population‐based case‐control study. Oncotarget. 2018;9(1):881‐891. 29416663 10.18632/oncotarget.22971 PMC5787520

[49] Er LK , Wu S , Cheng T , Ko YL , Teng MS . Genome‐wide association study on Adiponectin‐mediated suppression of HDL‐C levels in Taiwanese individuals identifies functional haplotypes in CDH13. Genes (Basel). 2021;12(10):1582. 34680977 10.3390/genes12101582 PMC8535967

[50] Wan JP , Zhao H , Li T , Li CZ , Wang XT , Chen ZJ . The common variant rs11646213 is associated with preeclampsia in Han Chinese women. PLoS One. 2013;8(8):e71202. 23976997 10.1371/journal.pone.0071202 PMC3747203

[51] Vargas‐Alarcon G , Martinez‐Rodriguez N , Velazquez‐Cruz R , et al. The T>a (rs11646213) gene polymorphism of cadherin‐13 (CDH13) gene is associated with decreased risk of developing hypertension in Mexican population. Immunobiology. 2017;222(10):973‐978. 27682011 10.1016/j.imbio.2016.09.004

