# Supplementary material for: Adiponectin and the risk of gastrointestinal cancers in East Asians: Mendelian randomization analysis
Source: Cancer Med. 2022 Apr 5;11(12):2397–404. doi: 10.1002/cam4.4735 (PMC9189470; doi:10.1002/cam4.4735)

**Supplement**

**Adiponectin and the risk of gastrointestinal cancers in East Asians: Mendelian randomization analysis**

Hua Jiang, Daojun Hu, Jun Wang, Bo Zhang, Chiyi He, Jiyu Ning

TABLE S1. The numbers of case and controls and SNPs used in each GWAS for six gastrointestinal cancers.

| Outcomes | Number of participants | | Number of SNPs |
| --- | --- | --- | --- |
|  | Case | Control# |  |
| Esophageal cancer | 1300 | 195,745 | 8,885,106 |
| Gastric cancer | 6563 | 195,745 | 8,885,324 |
| Pancreatic cancer | 442 | 195,745 | 8,885,075 |
| Hepatocellular carcinoma | 1866 | 195,745 | 8,885,115 |
| Biliary tract cancer | 339 | 195,745 | 8,885,064 |
| Colorectal cancer | 7062 | 195,745 | 8,885,369 |

#, All of the controls selected for the GWAS were free of any type of cancer.

TABLE S2. Genetic instrumental tools used in this study.

| CHR | Position | SNP id | Effect allele | Other allele | eaf | Adiponectin | | | Esophageal cancer | | Gastric cancer | |
| --- | --- | --- | --- | --- | --- | --- | --- | --- | --- | --- | --- | --- |
|  |  |  |  |  |  | beta | se | P value | beta | se | beta | se |
| 16 | 82663288 | rs12051272 | T | G | 0.2875 | -0.4015 | 0.0187 | 1.82E-102 | -0.0036 | 0.0435 | 0.0375 | 0.0199 |
| 3 | 186549695 | rs10937273 | A | G | 0.404 | 0.1507 | 0.0154 | 1.05E-22 | 0.0177 | 0.0410 | -0.0295 | 0.0188 |
| 16 | 82642651 | rs11646213 | A | T | 0.8238 | -0.1943 | 0.0207 | 5.93E-21 | -0.0202 | 0.0524 | -0.0100 | 0.0239 |
| 16 | 82635368 | rs8062637 | T | C | 0.7037 | -0.1419 | 0.0164 | 4.40E-18 | 0.0246 | 0.0448 | 0.0320 | 0.0205 |
| 16 | 82625758 | rs16957806 | T | C | 0.4469 | -0.1006 | 0.015 | 1.79E-11 | -0.0577 | 0.0404 | 0.0305 | 0.0184 |
| 10 | 122945086 | rs3943077 | A | G | 0.5671 | 0.0934 | 0.0154 | 1.22E-09 | -0.0363 | 0.0407 | 0.0023 | 0.0186 |
| 16 | 82601311 | rs16957722 | A | G | 0.8283 | -0.1121 | 0.0201 | 2.40E-08 | -0.0030 | 0.0534 | 0.0614 | 0.0244 |
| 16 | 82558174 | rs3843721 | T | C | 0.2002 | -0.1111 | 0.02 | 2.72E-08 | -0.0585 | 0.0564 | -0.0055 | 0.0257 |

Continued

| Pancreatic cancer | | Hepatocellular carcinoma | | Biliary tract cancer | | Colorectal cancer | |
| --- | --- | --- | --- | --- | --- | --- | --- |
| beta | se | beta | se | beta | se | beta | se |
| -0.0506 | 0.0741 | -0.0399 | 0.0365 | -0.1449 | 0.0848 | 0.0053 | 0.0191 |
| -0.0409 | 0.0701 | 0.0187 | 0.0344 | 0.0673 | 0.0802 | -0.0040 | 0.0180 |
| 0.0907 | 0.0897 | -0.0871 | 0.0440 | -0.2060 | 0.1025 | -0.0298 | 0.0230 |
| -0.0492 | 0.0769 | -0.0525 | 0.0376 | -0.0624 | 0.0879 | 0.0024 | 0.0197 |
| 0.0140 | 0.0688 | -0.0450 | 0.0339 | 0.0853 | 0.0788 | 0.0167 | 0.0177 |
| -0.0408 | 0.0695 | 0.0241 | 0.0342 | -0.0221 | 0.0795 | 0.0243 | 0.0179 |
| -0.0380 | 0.0917 | -0.1039 | 0.0448 | -0.0416 | 0.1049 | -0.0056 | 0.0235 |
| -0.0584 | 0.0958 | -0.0342 | 0.0473 | -0.1419 | 0.1095 | 0.0373 | 0.0247 |

eaf: frequency of effect allele.

TABLE S3. The associations between adiponectin and gastrointestinal cancers according to Mendelian randomization analysis.

| Outcome | Method | Odds ratio | 95% CI | |
| --- | --- | --- | --- | --- |
| Esophageal cancer | Inverse variance weighted | 1.05 | 0.89 | 1.23 |
|  | MR-Egger | 0.97 | 0.71 | 1.34 |
|  | Weighted mode | 1.03 | 0.84 | 1.26 |
|  | Weighted median | 1.02 | 0.84 | 1.23 |
| Gastric cancer | Inverse variance weighted | 0.88 | 0.81 | 0.96 |
|  | MR-Egger | 0.95 | 0.80 | 1.12 |
|  | Weighted mode | 0.90 | 0.82 | 0.99 |
|  | Weighted median | 0.89 | 0.82 | 0.98 |
| Pancreatic cancer | Inverse variance weighted | 1.04 | 0.78 | 1.37 |
|  | MR-Egger | 1.18 | 0.69 | 2.04 |
|  | Weighted mode | 1.15 | 0.80 | 1.64 |
|  | Weighted median | 1.08 | 0.77 | 1.50 |
| Hepatocellular carcinoma | Inverse variance weighted | 1.26 | 1.09 | 1.44 |
|  | MR-Egger | 1.00 | 0.76 | 1.30 |
|  | Weighted mode | 1.12 | 0.94 | 1.34 |
|  | Weighted median | 1.13 | 0.96 | 1.34 |
| Biliary tract cancer | Inverse variance weighted | 1.54 | 1.12 | 2.12 |
|  | MR-Egger | 1.80 | 0.97 | 3.36 |
|  | Weighted mode | 1.52 | 1.01 | 2.28 |
|  | Weighted median | 1.49 | 1.02 | 2.19 |
| Colorectal cancer | Inverse variance weighted | 1.00 | 0.93 | 1.07 |
|  | MR-Egger | 1.00 | 0.86 | 1.16 |
|  | Weighted mode | 0.99 | 0.90 | 1.09 |
|  | Weighted median | 0.99 | 0.90 | 1.08 |

FIGURE S1. Leave-one-out analysis for esophageal cancer.


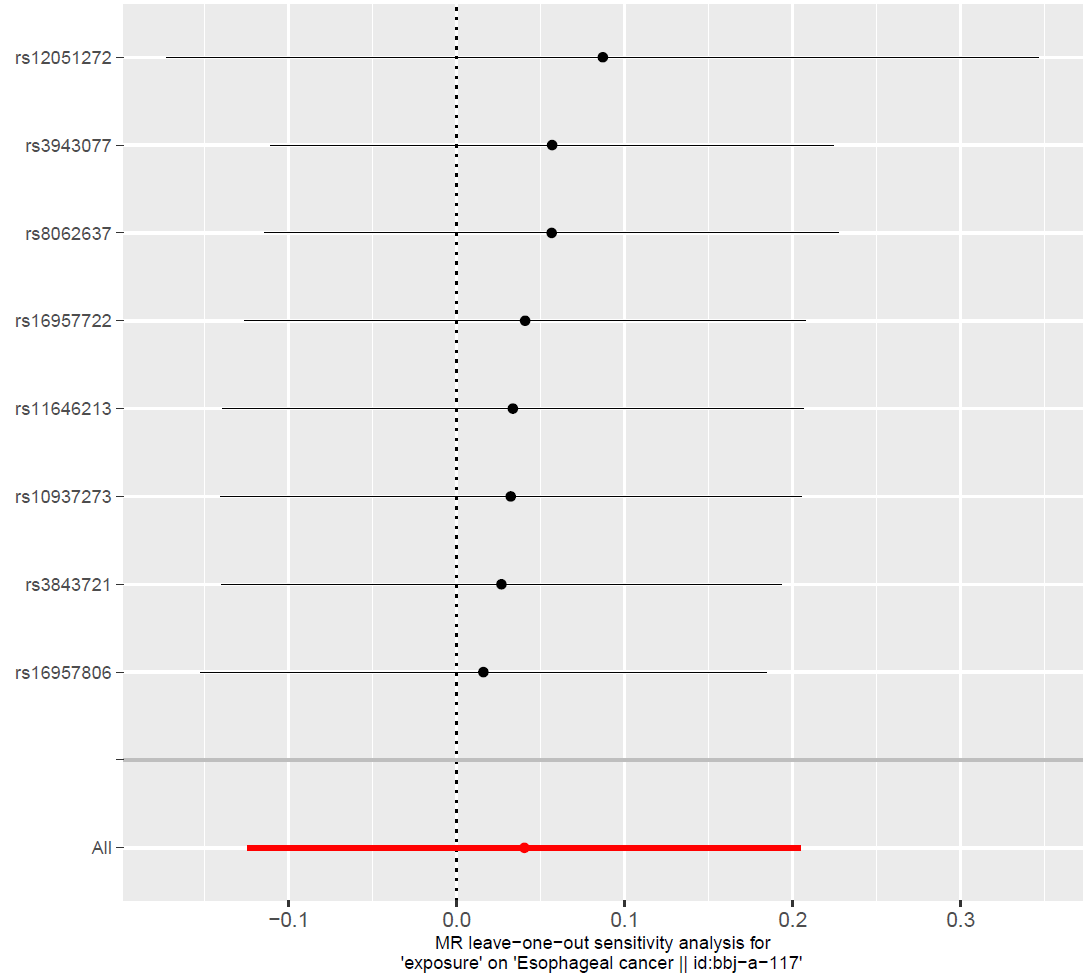


FIGURE S2. Leave-one-out analysis for gastric cancer.


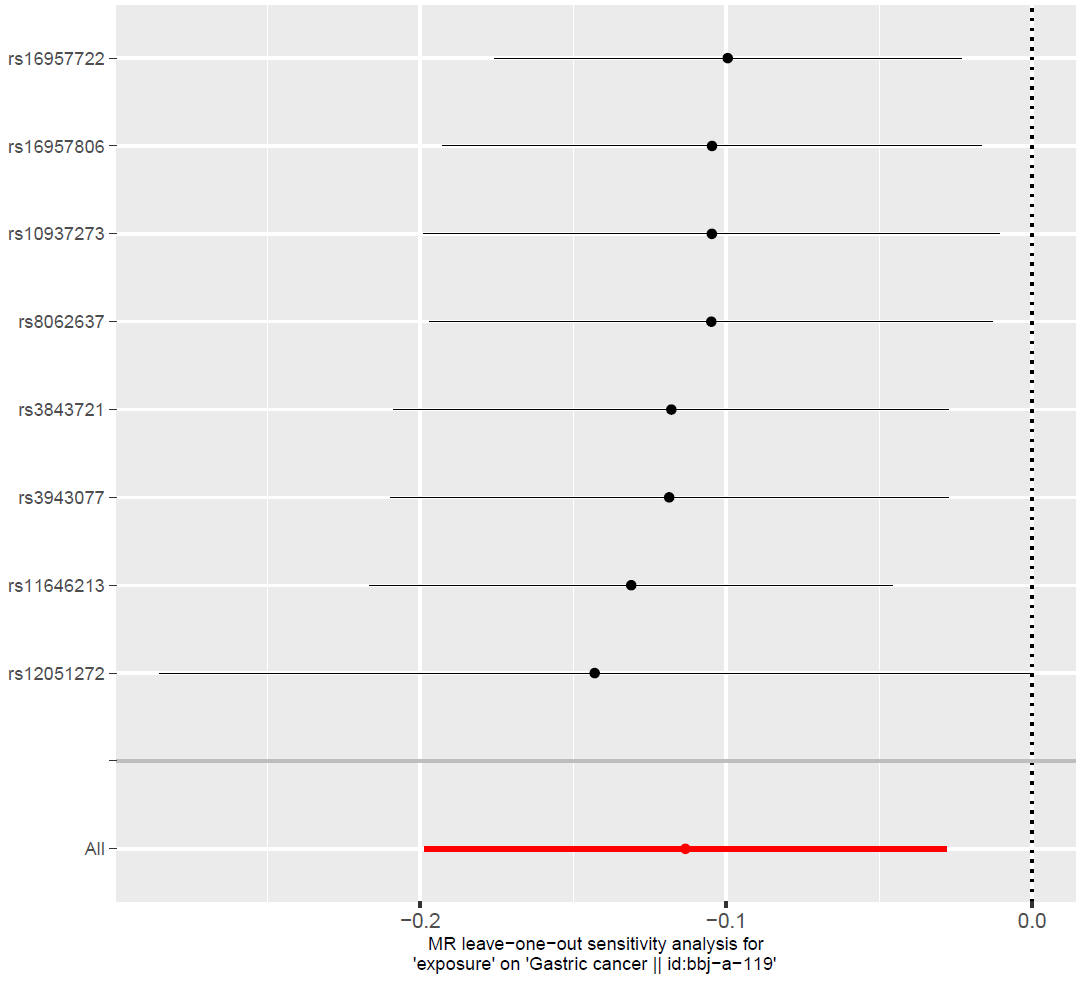


FIGURE S3. Leave-one-out analysis for pancreatic cancer.


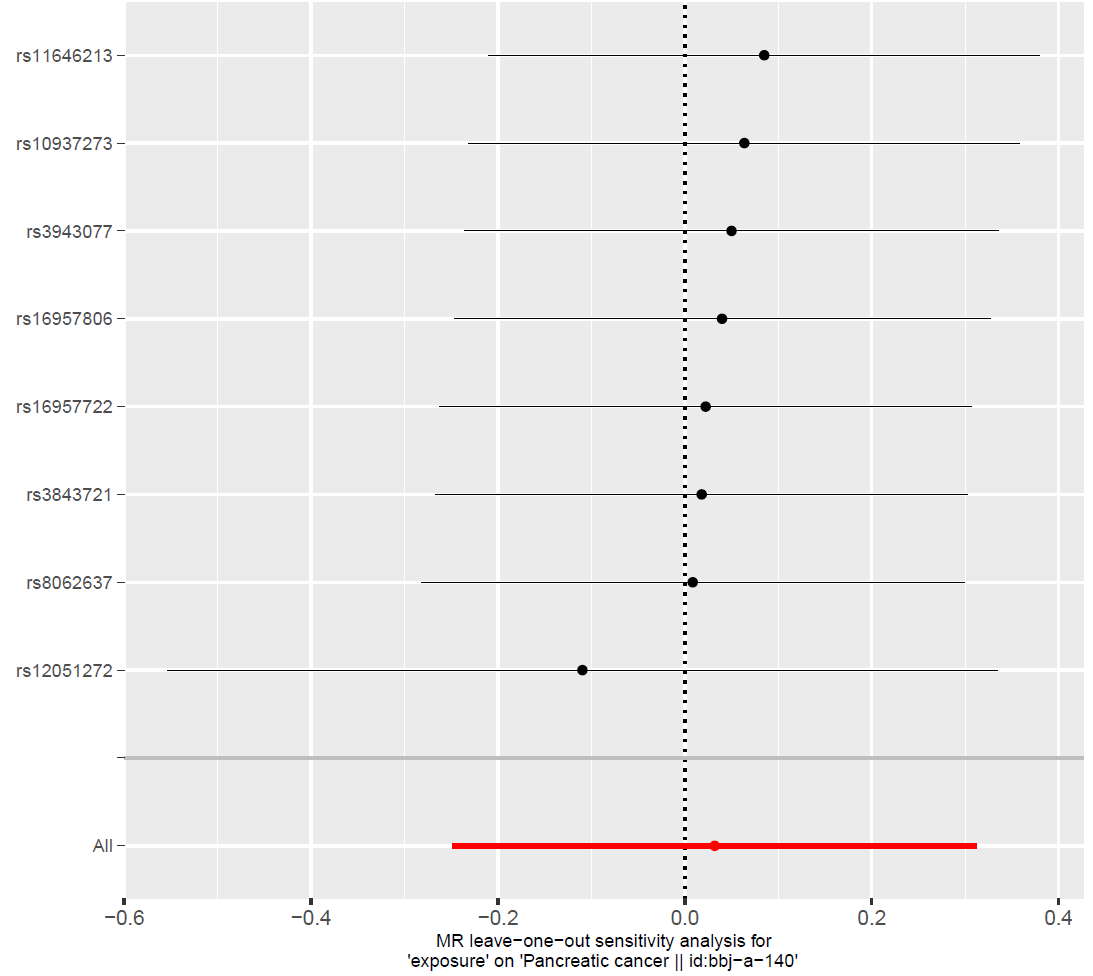


FIGURE S4. Leave-one-out analysis for hepatocellular carcinoma.


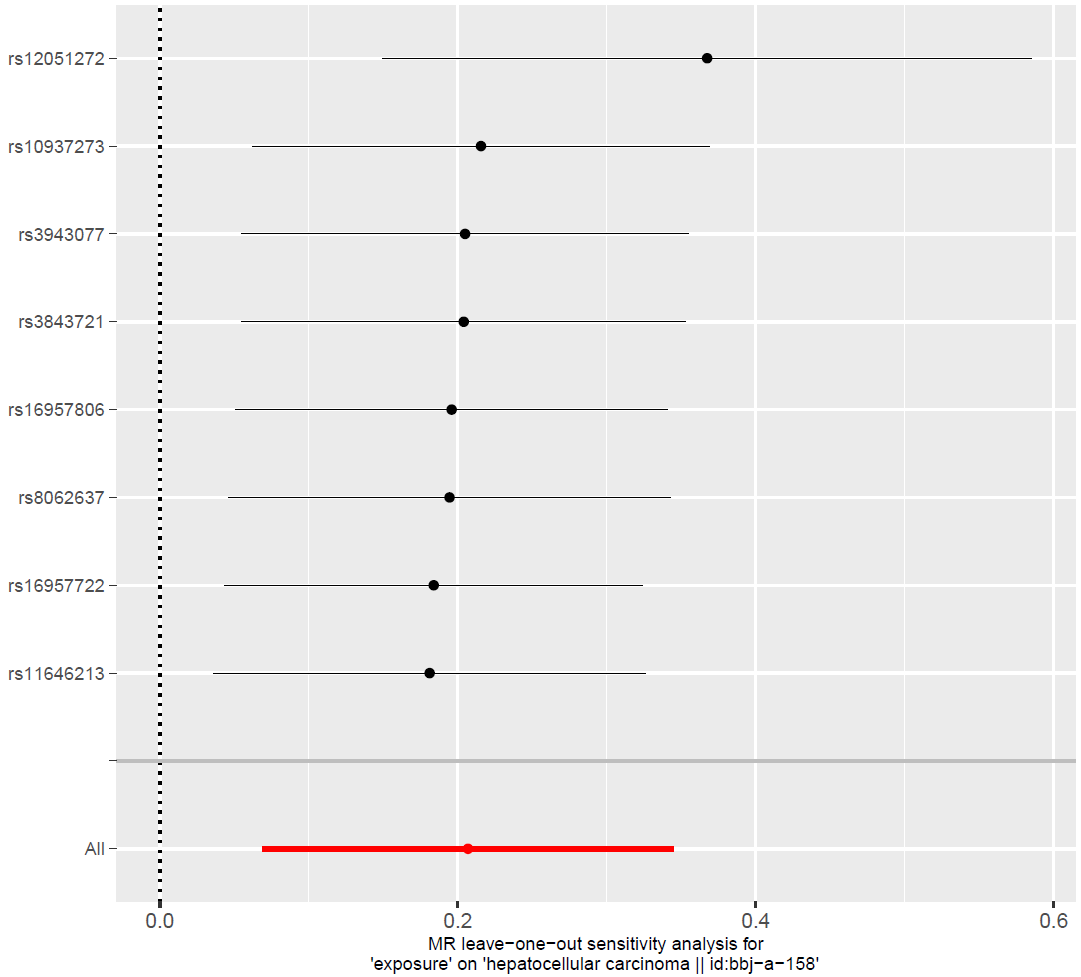


FIGURE S5. Leave-one-out analysis for biliary tract cancer.


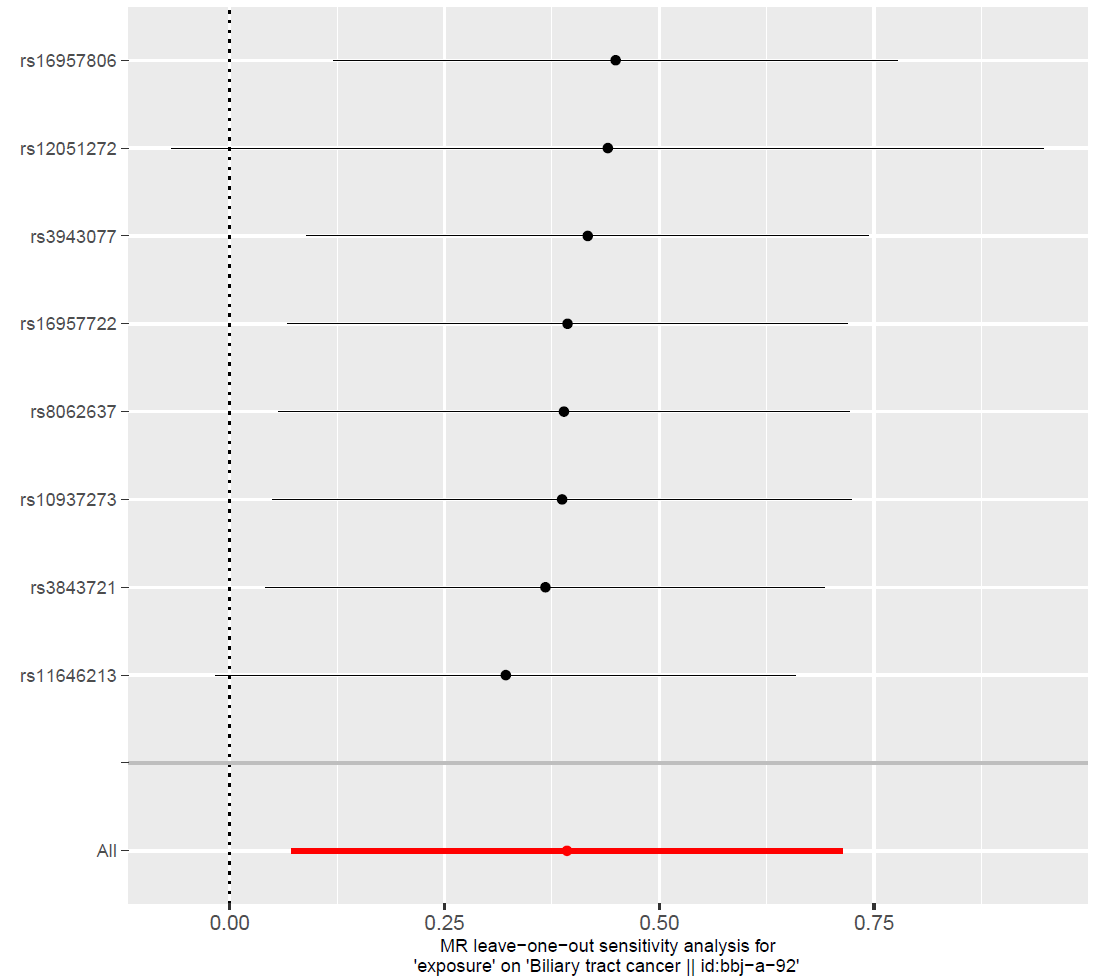


FIGURE S6. Leave-one-out analysis for colorectal cancer.


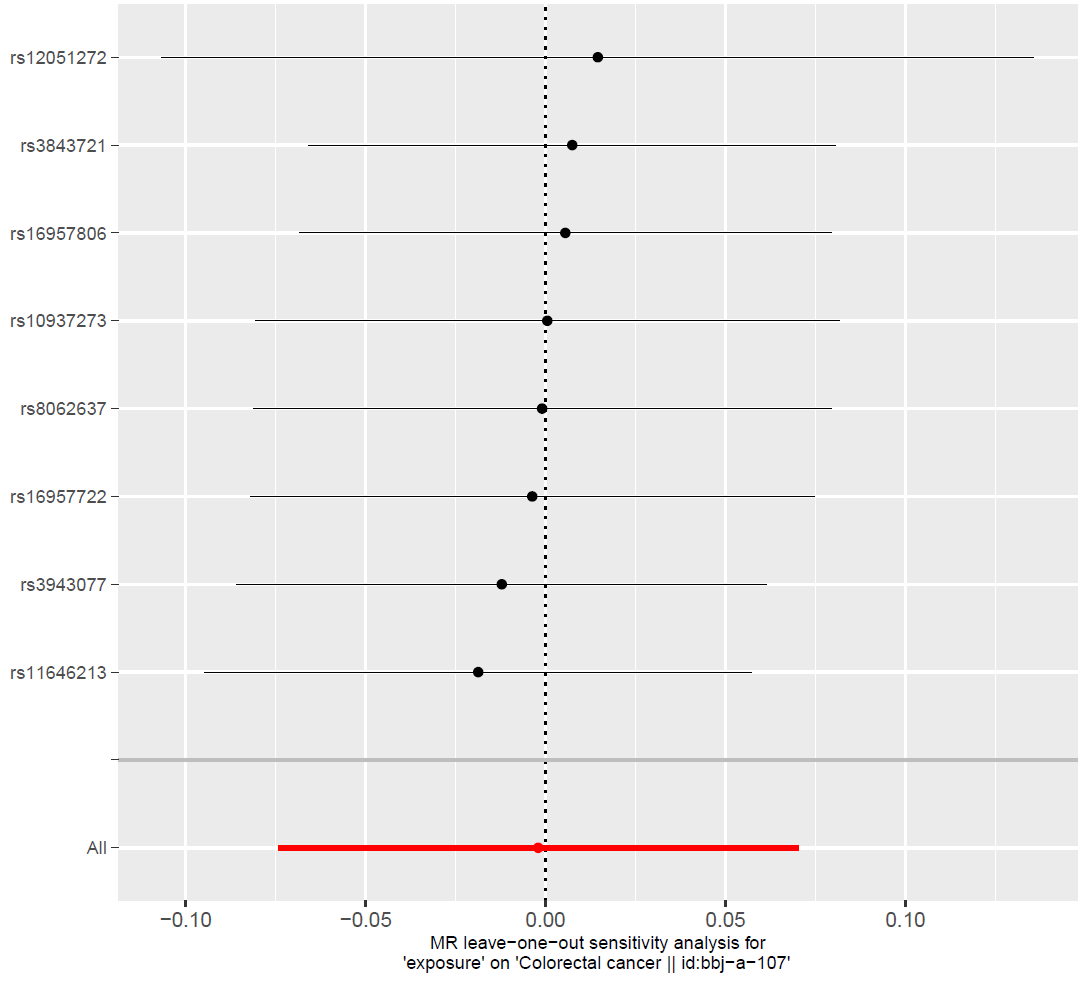

Supplement: Supplementary file 1 — Appendix S1 [file CAM4-11-2397-s001.docx]
